# Genomic characterization of multi drug resistant ESBL-producing *Escherichia coli* isolates from patients and patient environments in a teaching hospital in Ghana

**DOI:** 10.1186/s12866-024-03406-1

**Published:** 2024-07-08

**Authors:** Esther Eyram Asare Yeboah, Nicholas Agyepong, Joshua Mbanga, Daniel Gyamfi Amoako, Akebe Luther King Abia, Arshad Ismail, Alexander Owusu-Ofori, Sabiha Yusuf Essack

**Affiliations:** 1 Antimicrobial Research Unit, College of Health Sciences, University of KwaZulu-Natal, Durban, South Africa; 2 Department of Pharmaceutical Sciences, School of Pharmacy, Central University, P.O.Box 2305, Miotso, Ghana; 3 Department of Pharmaceutical Sciences, Sunyani Technical University, Sunyani, Ghana; 4 Department of Applied Biology & Biochemistry, National University of Science and Technology, P Bag AC939, Bulawayo, Zimbabwe; 5 Department of Integrative Biology and Bioinformatics, University of Guelph, Guelph, ON Canada; 6 Environmental Research Foundation, Westville, 3630 South Africa; 7 Sequencing Core Facility, National Institute for Communicable Diseases, National Health Laboratory Service, Johannesburg, 2131 South Africa; 8 Department of Biochemistry and Microbiology, Faculty of Science, Engineering and Agriculture, University of Venda, Tohoyandou, 0950 South Africa; 9 Department of Clinical Microbiology, School of Medicine and Dentistry, Kwame Nkrumah University of Science and Technology, Kumasi, Ghana; 10 Clinical Microbiology Unit, Laboratory Services Directorate, Komfo Anokye Teaching Hospital, Kumasi, Ghana

**Keywords:** Escherichia coli, Extended spectrum β-lactamase, Genomics, Mobile genetic elements, Phylogeny

## Abstract

**Background:**

ESBL-producing *Escherichia coli* pose a growing health risk in community and healthcare settings. We investigated the resistome, virulome, mobilome, and genetic relatedness of multidrug-resistant (MDR) *E. coli* isolates from patients and their environment in a Ghanaian teaching hospital.

**Materials and methods:**

Twenty-three MDR ESBL-producing or carbapenem-resistant *E. coli* isolates from a collection of MDR Gram-negative bacteria (GNB) from patients and environments were selected for genomic analyses. Whole genome sequencing and bioinformatics tools were used to analyze genomic characteristics and phylogeny.

**Results:**

The prevalence and incidence of rectal carriage of ESBL *E. coli* among patients were 13.65% and 11.32% respectively. The β-lactamase genes, *bla*_TEM−1B_ (10 isolates) and *bla*_CTX−M−15_ (12 isolates) were commonly associated with IncFIB plasmid replicons and co-occurred with aminoglycoside, macrolide, and sulfamethoxazole/trimethoprim resistance. Insertion sequences, transposons, and class I integrons were found with *bla*_CTX−M−15_. Carriage and environmental isolates carried multiple virulence genes, with *ter*C being the most prevalent in 21 isolates. Seventeen sequence types (STs) were identified, including a novel ST (ST13846). Phylogenetic analysis grouped the isolates into four main clusters, with one outlier. High genetic relatedness was observed between two carriage isolates of ST940 and between a carriage isolate and an environmental isolate of ST648. Isolates with different STs, collected at different times and locations, also showed genetic similarities.

**Conclusion:**

We identified ESBL-producing *E. coli* with diverse genomic characteristics circulating in different hospital directorates. Clonal relatedness was observed among isolates from patients and the environment, as well as between different patients, suggesting transmission within and between sources.

**Supplementary Information:**

The online version contains supplementary material available at 10.1186/s12866-024-03406-1.

## Introduction

In low- middle income (LMIC) countries, antimicrobial resistance is a problem leading to high death rates and is further exacerbated by the sub-optimal surveillance and poor infection prevention and control practices [1]. Multi-drug resistant Gram-negative bacteria (MDR GNB) have been implicated in critical infections in clinical settings [2].

*Escherichia coli* has the ability to colonize and persist in hosts and in the environment [3]. Hospitalised patients may harbour *E. coli* obtained from the community or acquire it after admission from various sources including the hospital environment via healthcare workers hands or being in close contact with other patients habouring resistant isolates or by touching contaminated surfaces [4, 5]. *E. coli* isolates may carry a repertoire of resistance and virulence genes carried on mobile genetic elements (MGEs). These MGEs include plasmids, integrons and insertion sequences which may be exchanged by horizontal gene transfer [6–8].

Extended spectrum β-lactamase (ESBL)–producing *Escherichia coli* which cause critical infections such as pneumonia and bloodstream infections are listed among the WHO priority organisms for which new antibiotics are required [9]. Colonization by antibiotic resistant organisms usually precedes infections with the same organisms thus the intestinal carriage of ESBL *E. coli* is a threat to health as infections caused by these result in poor treatment outcomes and high mortalities, especially among high-risk patients [5, 10]. It is estimated that globally, between 2003 and 2018, the cumulative global pooled prevalence of ESBL *E. coli* intestinal carriage in the community was 16.5% [5]. Colonization of hospital patients with ESBL producing *E. coli* isolates have been reported in African countries such as South Africa and Tanzania [11, 12]. Recent studies in a Ghanaian hospital reported that about 13.2% of diarrhoegenic *E. coli* isolates from patients were ESBL positive and cefotaxime resistant ESBL *E. coli* was found to be widely disseminated in Ghanaian hospitals [13, 14].

Patients may be colonized on admission to hospitals and could be a potential source of transfer to other patients who could become infected in hospitals [10]. Resistance and virulence genes of ESBL *E. coli* isolates from patients could be shed into the hospital environment through the skin, respiratory or intestinal tract and further transferred to other patients in hospitals.

*E. coli* isolates showing varying resistance to β-lactams have been studied in clinical settings in Ghana but most of these have been isolates from infected patients with limited studies on the molecular epidemiology of these MDR *E. coli* isolates. There are also limited studies on colonization with ESBL *E. coli* and their potential transmission to other patients and environment, particularly in non-outbreak settings. We investigated the molecular epidemiology of MDR ESBL-producing *E. coli* colonizing patients, carried on healthcare workers hands and contaminating selected ward and intensive care unit (ICU) environments. This was done to inform infection prevention and control (IPC) measures so as to control their spread in clinical settings.

## Materials and methods

### Study setting

This study was conducted at the Komfo Anokye Teaching hospital (KATH) which is 1200 - bed capacity government tertiary facility which serves as a referral hospital responding to the healthcare needs of about 80% of both emergencies and regular medical cases in the Ashanti region of Ghana that has a population of about 5.4 million. The hospital also attends to referral cases from other regions including the Bono, Bono East, Ahafo, Western and Eastern regions as well as some parts of the Northern regions of the country. This study was conducted in three directorates; Obstetrics and Gynaecology, Surgery and the Intensive Care Unit (ICU).

### Sample collection

Samples were collected from consenting patients admitted to the ICU and Surgery, and Obstetrics and Gynaecology directorates of the hospital. Rectal swabs and hand swabs were aseptically collected from in-patients > 18 years old, at admission and after 48 h as previously described in the preceding study. Isolation of Gram-negative bacteria and antibiotic susceptibility testing was carried out as previously described [15].

### DNA extraction and whole genome sequencing

All MDR ESBL- producing or carbapenem resistant *E. coli* isolates were subjected to genomic DNA (gDNA) extraction using the GenElute^®^ bacterial genomic DNA kit (Sigma-Aldrich, St. Louis, MO, United States) according to the manufacturer’s instructions. ESBL-*E. coli* were defined as isolates with phenotypic resistance to at least one the third generation cephalosporins on VITEK. The quantity and quality of the extracted gDNA were determined using a Nanodrop spectrophotometer Qubit (Thermo Scientific, Waltham, MA, USA). Multiplexed paired-end libraries (2 × 300 bp) were prepared using the Nextera XT DNA sample preparation kit (Illumina, San Diego, CA, United States), and sequences were determined on an Illumina Nextseq 550 (2 × 150 bp) platform with 100× coverage.

### Genomic analyses and annotation

Quality trimming of raw reads was done using Sickle v1.33 (https://github.com/najoshi/sickle). The raw reads were then assembled spontaneously using the SPAdes v3.6.2 assembler (https://cab.spbu.ru/software/spades/). All contiguous sequences were subsequently submitted to GenBank and assigned accession numbers under BioProject **PRJNA823741** (Supplementary Table S1).

Multilocus sequence typing (MLST) of assembled genomes was determined on the MLST 1.8 database hosted by the Center for Genomic Epidemiology (CGE) (https://cge.food.dtu.dk/services/MLST/). Isolates with unknown STs were submitted to the EnteroBase *Escherichia*/*Shigella* database (https://enterobase.warwick.ac.uk/species/index/ecoli) and assigned novel STs. Resistance and virulence genes were determined using ResFinder (https://cge.food.dtu.dk/services/ResFinder/) and Virulence finder (https://cge.food.dtu.dk/services/VirulenceFinder/), and plasmids by PlasmidFinder 2.1 hosted on https://cge.food.dtu.dk/services. INTEGRALL (http://integrall.bio.ua.pt/) and RAST SEEDVIEWER (https://rast.nmpdr.org/seedviewer.cgi) were used to find integrons and transposons. Insertion sequences, and prophages were determined using ISFinder (https://isfinder.biotoul.fr/) and PHASTER (https://phaster.ca/), respectively. Plasmids of the IncF, IncH1, IncH2, IncI1, IncN, or IncA/C types were subtyped by assigning a replicon allele at the plasmid MLST site (https://pubmlst.org/plasmid/). Phylogroups were determined via ClermonTyper (http://clermontyping.iame-research.center/*).*

The synteny and genetic environment of antibiotic resistance genes (ARGs) and associated MGEs was investigated using GenBank’s general feature format (GFF3) files. The genetic environment of virulence genes detected in the study was also determined using a similar approach.

### Phylogenomics

Phylogenomic analysis was undertaken to determine how the study isolates compare to *E. coli* genomes of human origin from South Africa and West African countries including Togo, Nigeria, Niger, Mali, Ghana, and Cameroon. All the *E. coli* genomes were reported in these countries from 2013 to 2021 (*n* = 157). The genomes were downloaded from the Bacterial and Viral Bioinformatics Resource Center (BV-BRCB) website (https://www.bv-brc.org/), annotated (Table S1), and included in the analysis. The phylogenetic tree was constructed based on the maximum likelihood method using BV-BRCB. The *Escherichia coli* K12-MG1655 was used as the outgroup strain (reference genome), facilitating the configuration of the phylogenetic distance between the isolates on the branches. The Figtree software (https://tree.bio.ed.ac.uk/software/figtree/) and iTOL (https://itol.embl.de/) were used to visualize, edit, and annotate the generated phylogenetic tree.

Phandango (https://jameshadfield.github.io/phandango/#/main), was used to visualise the phylogenetic tree with corresponding metadata to get a more comprehensive insight on the relationships of the isolates.

## Results

### Isolate characteristics

ESBL *E. coli* was isolated from 17 patients. Eleven of 83 (13.25%) patients were colonized with ESBL *E. coli* on admission. ESBL *E. coli* was also isolated from a patient’s hand on admission. Six of 53 (11.32%) patients who had samples taken after 48 h of admission, had acquired ESBL *E. coli.* Of the 208 environmental samples taken, seven (3.37%) were contaminated with ESBL *E. coli*. None of the healthcare workers (HCW’s) hands were contaminated with ESBL *E. coli*. Of the seven environmental samples with ESBL *E. coli*, four were isolated from beds, two from taps and one from a dripstand.

Twenty-three MDR *E. coli* isolates which were both MDR and ESBL-producing (21 isolates) or MDR carbapenem resistant (two isolates) *E. coli* isolates were subjected to WGS. Sixteen were from patients, and seven were from the hospital environment. All the carriage *E. coli* isolates were obtained from rectal swabs except one that was obtained from a hand swab. No isolates were obtained from healthcare workers’ hands. Three of the environmental isolates were obtained from the surgical ward, and the remaining from the obstetrics and gynaecology ward (Table 1).

### Antimicrobial susceptibility of studied isolates

All of the MDR *E. coli* isolates selected were susceptible to amikacin, imipenem, and tigecycline. Highest resistance was observed against ampicillin (100%), sulphamethoxazole/trimethoprim (21/23, 91.3%), ceftazidime (21/23, 91.3%), cefuroxime (20/23, 87%), amoxicillin/clavulanic (21/23, 91.3%). Ten (23.3%) of the isolates were resistant to the carbapenems; doripenem, ertapenem and meropenem. Six isolates from patients and four from hospital environment were resistant to ciprofloxacin. A total of nine resistance profiles were observed across the 23 selected *E. coli* isolates. The most common resistance pattern (AMP-CXM-CAZ-CRO-FEP-GEN-TOB-AMC-TZP-CIP-SXT) was observed among nine *E. coli* isolates (five from the hospital environment and four from patients) (Table 1) suggesting the contamination of environmental surfaces with isolates of similar resistant patterns as those colonizing patients.

### Antibiotic resistance genes

The resistance genotypes corresponded to the phenotypic resistance in most of the isolates except for a few isolates where there were no identified genotypes corresponding to phenotypic resistance to antibiotics. In one isolate (P165) from a patient, there were no antibiotic resistance genes corresponding to its phenotypic resistance to AMP-CXM-CAZ-CRO-FEP-GEN-TOB-AMC-TZP-CIP-SXT.

ESBL genes were identified in 19 of the 23 isolates and mostly belonged to the CTX-M and TEM classes. The most common ESBL identified among the *E. coli* isolates was *bla*_CTX−M−15_ harboured by 11 isolates from patients and one environmental isolate. The *bla*_TEM−104_ and *bla*_TEM−169_ genes were each harboured by *E. coli* from a patient. The β-lactamase *bla*_OXA−1_ was detected in 12 isolates (nine carriage and three environmental isolates). The *bla*_TEM−1B_ gene was also detected in 10 isolates. The carbapenemase gene *bla*_OXA−181_ was detected in two isolates from patients. In one isolate (P2R), *bla*_OXA−181_ was detected even though there was no phenotypic expression of carbapenem resistance.

An environmental isolate, (E50-1) which showed phenotypic resistance to doripenem and meropenem had no carbapenemase or β-lactamase genes. Another isolate (P17) from a patient, harboured thirteen ESBLs (*bla*_CTX−M−22,_*bla*_CTX−M−216_, *bla*_CTX−M−103_, *bla*_CTX−M−176_, _*bla*CTX−M−15,_*bla*_CTX−M−156_, *bla*_CTX−M−3_, *bla*_CTX−M−202_, *bla*_CTX−M−88_, *bla*_CTX−M−203_, *bla*_CTX−M−71_, *bla*_CTX−M−167_, and *bla*_OXA−1_). The *bla*_TEM−1B_ and *bla*_CTX−M−15_ genes commonly occurred with sulphamethoxazole and trimethoprim-resistant genes *sul2* and *dfrA17*, respectively (Table 1).

Genes conferring resistance to aminoglycosides (*aadA1*,* aadA2*,* aadA5*,* aph(6)-Id*,* aac(6’)-Ib-cr*, *aph(3’’)-Ib*,* aac(3)-IId*), macrolides (*mphA* and *ermB*), tetracycline (*tetA*,* tetB*, *tet*L and *tetM*), sulphamethoxazole (*sul1*,* sul2*,* sul3*) and trimethoprim (*dfrA1*,* dfrA7*,* dfrA12*,* dfrA14*,* dfrA17 and dfrG*) were also identified in both patient and environmental isolates. The plasmid-mediated quinolone resistant (PMQR) genes, *aac(6’)-Ib-cr*,* qnrS1*,* qnrB19*,* qnrB4* and *qepA4* were also detected in the isolates. The quaternary ammonium compound resistance genes, *qacE*, were found in eight isolates (seven from patients and one from environmental isolates).

Reduced susceptibility to ciprofloxacin was observed in 10 isolates (six carriage isolates and four environmental isolates), therefore, mutations in the quinolone resistance determinant regions (QRDRs) DNA gyrase (*gyrA* and *gyrB*) and DNA topoisomerase IV (*parC* and *parE*) genes in the isolates from patients and environments were investigated. Mutations were commonly found in *gyrA* (S83L, D87N, A828S, D678E, A863V) and *parC* (E62K, S801, D475E, S80I, L440R) genes, with *gyrB* (S492N, A618T, E656D, E703D) and *parE* (T172A, S458A) having the least number of mutations. These mutations were commonly associated with other Plasmid mediated quinolone resistance (PMQR) determinants such as *qnr*. Nine isolates (four from environment and five from patients) had common mutations in *gyr A* (S83L, D87N), *parC* (S80I) and *parE* (S458A). One isolate (P51B) from a patient had mutations in all four genes: *gyrA* (S83L, D87N, A828S, D678E),* gyrB (S492N*,* A618T*,* E656D)*,* parC (E62K*,* S801*,* D475E)* and *parE (T172A*,* S458A)*(Table S3).

**Table 1:** Source, antibiograms, sequence types (STs), phylogroups, resistance genes, virulence genes and plasmids found in the *Escherichia coli* isolates

ID	Source	Resistance pattern	Sequence Type	Phylogroup	ABR genes		Virulence genes	Plasmid replicon	Pmlst
					β-lactamases	Others			
P51B	Patient	AMP-CXM-CAZ-CRO-FEP-AMC-TZP-GEN-TOB-CIP-SXT	ST648	F	*bla*_*TEM−1B*_, *bla*_*CTX−M−15*_, *bla*_*OXA−1*_	*sul2*,* dfrA17*,* tet(B)*,* tet(M)*,* mph(A)*,* aac(3)-IIa*,* aac(6’)-Ib-cr*	*air*,* astA*,* chuA*,* eilA*,* fyuA*,* gad*,* hlye*,* hra*,* irp2*,* kpsM*,* kpsE*,* lpfA*,* papA*,* papC*,* terC*,* traT*,* yfcv*	Col(BS512), IncFIA, IncFIB(pB171), IncFII, IncI1-I(Alpha), IncQ1, IncX1	IncF[F2:A1:B32]
E53	Environment (Bed)	AMP-CXM-CAZ-CRO-FEP-AMC-TZP-GEN-TOB-CIP-SXT	ST648	F	*bla*_*OXA−1*_, *bla*_*TEM−169*_, *bla*_*CTX−M−15*_, *bla*_*TEM−33*,_	*sul2*,* dfrA17*,*aac(6’)-Ib-cr*,* aac(3)-IId*,* mph(A)*,* erm(B)*,* tet(M)*,* tet(B)*	*terC*,* ipfA*,* papC*,* yfcV*,* papa_F43*,* eilA*,* hra*,* chuA*,* irp2*,* fyuA*,* astA*,* gad*,* kpsMIII_K98*,* traT*,* kpsE*	Col(BS512), IncFIA, IncFIB(pB171), IncFII, IncQ1, IncX1	IncF[F2:A1:B32]
P2R	Patient	AMP-CXM-CAZ-CRO-FEP-AMC-TZP-SXT	ST940	B1	*bla*_*TEM−35*_, *bla*_*OXA−181*_	*sul2*,* dfrA12*,* dfrA1*,* aph(6)-Id*,* aadA2*,* aph(3’’)-Ib*,* qnrS1*,*erm(B)*,* tet(B)*	*lpfA*,* gad*,* hlyE*,* traT*,* capU*,* terC*	IncFIA, IncFIB, IncFIC(II), ColKP3	IncF [F-:A-:B12]
P73	Patient	AMP-CXM-CAZ-CRO-FEP-MEM-AMC	ST940	B1	*bla*_*OXA−1*_, *bla*_*OXA−181*_, *bla*_*TEM−35*_	*sul2*,* aph(3’’)-Ib*,* aph(6)-Id*,* aadA1*,*qnrS1*,* tet(B)*	*lpfA*,* gad*,* traT*,* hlyE*,* iss*,* capU*,* terC*	ColKP3, IncFIA, IncFIB, IncFII	IncF[F36:A1:B1]
P128	Patient	AMP-CAZ-CRO-FEP-AMC-TZP-SXT	ST13846	D	*bla*_*TEM−1B*_, *bla*_*CTX−M−15*_	*sul2*,* dfrA14*,* aph(6)-Id*,* aph(3’’)-Ib*,* qnrS1*,* tet(A)*	*ipfA*,* eilA*,* irp2*,* chuA*,* sitA*,* fyuA*,* gad*,* kpsMIII_K96*,* ompT*,* iss*,* kpsE*,* terC*	IncY	
P166	Patient	AMP-CXM-CAZ-CRO-FEP-AMC-TZP-SXT	ST13846	D	*bla*_*TEM−1B*_, *bla*_*CTX−M−15*_	*sul2*,* dfrA14*,* aph(6)-Id*,* aph(3’’)-Ib*,* qnrS1*,* tet(A)*	*ipfA*,* air*,* eilA*,* sitABCD*,* irp2*,* chuA*,* fyuA*,* gad*,* kpsMIII_K96*,* ompT*,* kpsE*,* terC*	IncY	
P49	Patient	AMP-CXM-CAZ-CRO-FEP-AMC-TZP-ERT-SXT	ST73	B2	*bla*_*TEM−1B*_	*sul1*,* sul2*,* dfrA1*,* dfrA17*,* dfrG*,* aadA5*,* aadA1*,* aph(3’’)-Ib*,* aph(6)-Id*,* erm(B)*,* tet(B)*,* tet(M)*,* tet(L)*,* qacE*	*ccI*,* cea*,* clbB*,* cnf1*,* focc*,* focG*,* hlyA*,* hra*,* ibeA*,* ireA*,* iroN*,* iss*,* iucC*,* iutA*,* kpsE*,* kpsM*,* mchB*,* mchf*,* mchC*,* mcmA*,* neuc*,* ompT*,* papA*,* papC*,* pic*,* senB*,* sfaD*,* sitA*,* tcpC*,* terC*,* traT*,* usp*,* vat*	Col(MG828), Col156, ColRNAI, IncFIA, IncFIB, IncFII, IncQ1	IncF[F36:A1:B1]
P142	Patient	AMP-CXM-CAZ-CRO-FEP-AMC-TZP-DOR-MEM-SXT	ST5614	B1	*bla*_*TEM−104*_, *bla*_*TEM−1B*_, *bla*_*TEM−198*_, *bla*_*CTX−M−15*_, *bla*_*TEM−217*_, *bla*_*TEM−234*_	*sul1*,* sul2*,* dfrA1*,* dfrA17*,*aph(3’’)-Ib*,* aadA5*,* aph(6)-Id*,* qnrS1*,* mph(A)*,* tet(A)*,* tet(B)*,* qacE*	*iha*,* ipfA*,* sigA*,* iucC*,* iutA*,* cib*,* gad*,* traT*,* iss*,* capU*,* terC*,* hlyE*	IncB/O/K/Z, IncFII, IncQ1	IncF [F-:A-:B] *FII
P17	Patient	AMP-CXM-CAZ-CRO-FEP-AMC-TZP-TOB-CIP-SXT	ST224	B1	*bla*_*CTX−M−22*_, *bla*_*CTX−M−216*_, *bla*_*CTX−M−103*_, *bla*_*CTX−M−176*_, *bla*_*CTX−M−15*_, *bla*_*CTX−M−156*_, *bla*_*CTX−M−3*_, *bla*_*CTX−M−202*_, *bla*_*CTX−M−88*_, *bla*_*CTX−M−203*_, *bl*_*aCTX−M−71*_, *bla*_*CTX−M−167*_, *bla*_*OXA−1*_,	*sul1*,* sul3*,* dfrA12*,* dfrA17*,* aadA1*,* aadA2*,* aadA5*,* aac(6’)-Ib-cr*,* mph(A)*,* erm(B)*,* tet(A)*,* qacE*	*ipfA*,* sitABCD*,* iucC*,* sitA*,* iutA*,* gad*,* traT*,* terC*	IncFIA, IncFIB, IncFII, IncI1-I(Alpha), IncY	IncF[F2:A4:B1]
P60R	Patient	AMP-CXM-CAZ-CRO-FEP-AMC-TZP-SXT	ST1722	F	*bla*_*CTX−M−15*_, *bla*_*TEM−1B*_	*sul2*,* dfrA14*,* aph(6)-Id*,* aph(3’’)-Ib*,* qnrS1*,* tet(A)*	*IpfA*,* afaA*,* afaB*,* afaC*,* afaD*,* air*,* eilA*,* cia*,* chuA*,* gad*,* traT*,* iss*,* kpsE*,* terC*	IncB/O/K/Z, IncI2(Delta), IncY, pXuzhou21	
P165	Patient	AMP-CXM-CAZ-CRO-FEP-AMC-TZP-GEN-TOB-CIP-SXT	ST3489	A	*-*		*neuC*,* gad*,* ompT*,* terC*	IncFIB(K), IncL, IncY	
P105	Patient	AMP-CXM-CAZ-CRO-FEP-AMC-TZP-GEN-TOB-CIP-SXT	ST10	A	*bla*_*OXA−1*_, *bla*_*CTX−M−15*_	*sul1*,* dfrA17*,* aadA5*,*aac(3)-IIa*,* aac(6’)-Ib-cr*,* mph(A)*,* tet(B)*,* qacE*	*hra*,* senB*,* sitABCD*,* iucC*,* irp2*,* sitA*,* iutA*,* fyuA*,* mchF*,* traT*,* terC*	IncFIA, IncFIB, IncFII	IncF[F36:A4:B1]
P159	Patient	AMP-CXM-CAZ-CRO-FEP-AMC-TZP-GEN-TOB -CIP-SXT	ST617	A	*bla*_*OXA−1*_, *bla*_*CTX−M−15*_	*sul1*,* sul2*,* dfrA17*,* aadA5*,* aph(6)-Id*,* aac(6’)-Ib-cr*,* aph(3’’)-Ib*,* mph(A)*,* tet(B)*,* qacE*	*sitABCD*,* iucC*,* irp2*,* sitA*,* iutA*,* fyuA*,* traT*,* iss*,* terC*	IncFIA, IncFIB	IncF[F-:A4:B1]
P72	Patient	AMP-CXM-CRO-CAZ-FEP-AMC-TZP-CIP-SXT	ST167	A	*bla*_*CTX−M−27*_	*sul1*,* sul2*,* dfrA12*,* aph(6)-Id*,* aph(3’’)-Ib*,* aadA2*,* qepA4*,* mph(A)*,* tet(A)*,* tet(B)*	*irp2*,* fyuA*,* traT*,* terC*	IncFIA, IncFIB, IncFII	IncF[F48:A1:B49]
E56	Environment (Bed)	AMP-CXM-CAZ-CRO-FEP-AMC-TZP-GEN-TZP-CIP-SXT	ST638	A	*bla*_*TEM−1B*_, *bla*_*OXA−1*_	*aac(3)-IIa*,* tet(A)*	*ipfA*,* yfcv*,* terC*	Col(BS512), IncFIB(K), IncR	
E37	Environment (Tap)	AMP-CXM-CAZ-CRO-FEP-AMC-TZP-GEN-TOB-CIP-SXT	ST410	C	*bla*_*CTX−M−15*_, *bla*_*OXA−1*_	*sul2*,* dfrA14*,*mph(A)*,* aac(3)-IIa*,* aac(6’)-Ib-cr*,* tet(B)*	*cba*,* cma*,* cnf1*,* fyuA*,* hlya*,* hlyE*,* hra*,* irp2*,* iucC*,* iutA*,* lpfA*,* papA*,* papC*,* sitA*,* terC*,* traT*	IncFIA, IncFIB, IncFII, IncQ1	IncF [F104:A1:B1]
E29	Environment (Tap)	AMP-CXM-CAZ-CRO-FEP-AMC-TZP-GEN-TOB-CIP-SXT	ST450	A	*bla*_*CTX−M−3*_, *bla*_*TEM−1B*_	*sul2*,* dfrA17*,*mph(A)*,* aac(3)-IId*,* tet(A)*	*fyuA*,* gad*,* hlyE*,* iha*,* irp2*,* iucC*,* iutA*,* kpsE*,* mchf*,* ompT*,* papA*,* papc*,* sat*,* senb*,* terC*,* traT*	Col(BS512), Col156, IncFIA, IncFIB, IncFII, IncI1-I(Alpha), IncI2(Delta)	IncF [F4:A2:B20]
E25B	Environment (Bed)	AMP-CXM-CAZ-CRO-FEP-AMC-TZP	ST127	B2	*bla*_*TEM−1D*_	*sul1*,* dfrA7*,*tet(B)*	*papC*,* sfaD*,* sfaE*,* sfaS*,* yfcv*,* papA*,* hra*,* vat*,* clbB*,* cnf1*,* tcpC*,* chuA*,* iroN*,* irp2*,* sitA*,* fyuA*,* usp*,* mcmA*,* gad*,* iss*,* kpsE*,* kpsMII_F48*,* ompT*,* traT*,* TerC*	IncFIA, IncFIB, IncFII	IncF[F1:A1:B20]
E50-1	Environment (Dripstand)	AMP-TZP-DOR-MEM-SXT	ST155	B1	*-*	*sul2*,* dfrA17*,* aadA5*,* tet(A)*	*ipfA*,* gad*,* ompT*,* iss*,* terC*	-	
E55-2	Environment (Bed)	AMP-DOR-MEM-SXT-	ST58	B1	*bla*_*TEM−1B*_	*sul1*,* sul2*,* dfrA17*,* aadA5*,* aph(3’’)-Ib*,* aph(6)-Id*,* qnrB19*,* mph(A)*,* tet(A)*,* qacE*	*iha*,* ipfA*,* papA_F43*,*sat*,* senB*,* iucC*,* sitABCD*,* iutA*,* cea*,* gad*,* traT*,* kpsMII_K52*,* iss*,* capU*,* terC*	Col(pHAD28), Col156, IncFIB, IncFI1(29)	IncF[F29:A-:B10]
P115	Patient	AMP-CXM-CAZ-CRO-FEP-AMC-TZP-SXT	Unknown	E	*bla*_*CTX−M−15*_, *bla*_*TEM−35*_, *bla*_*OXA−1*_	*sul2*,* dfrA1*,* drfA17*,* aadA1*,* aadA5*,* qnrS1*,* mph(A)*,* tet(B)*	*IpfA*,* papA_F19*,* papA_F20*,* eilA*,* V*,* terC*	Col(BS512), IncFIA, IncFIB, IncFII, IncFHI2, IncFHI2A	IncF[F36:A1:B20]
P143	Patient	AMP-CXM-CAZ-CRO-FEP-AMC-TZP-SXT	Unknown	Unknown	*bla*_*OXA−1*_	*sul2*,* dfrA14*,* tet(B)*	*eatA*,* irp2*,* fyuA*,* terC*	IncFII, IncL, IncN	IncF[[F-:A-:B-] FII_84*
P63	Patient	AMP-CXM-CAZ-CRO-FEP-AMC-TZP-GEN-TOB-CIP-SXT	Unknown	E	*bla*_*TEM−1B*_, *bla*_*OXA−1*_, *bla*_*CTX−M−15*_, *bla*_*DHA−1*_	*sul1*,* sul3*,* dfrA1*,* dfrA12*,* dfrA17*,* aac(6’)-Ib-cr*,* aac(3)-IIa*,* aadA2*,* aadA5*,* aadA1*,* qnrB4*,* mph(A)*,* erm(B)*,* tet(A)*,* tet(B)*	*ipfA*,* yfcv*,* iucC*,* irp2*,* sitABCD*,* iutA*,* fyuA*,* terC*	Col(BS512), IncFIA, IncFIB, IncFII, IncR	IncF[F36:A4:B1]

ampicillin (AMP), cefuroxime (CXM), ceftazidime (CAZ), ceftriaxone (CRO), cefepime (FEP), gentamicin (GEN), tobramycin (TOB), amikacin (AMK), amoxicillin/clavulanic (AMC), piperacillin/tazobactam (TZP), imipenem (IPM), doripenem (DOR), meropenem (MEM), ertapenem (ERT), ciprofloxacin (CIP), trimethoprim-sulphamethoxazole (SXT) and tigecycline (TGC)

### Mobilome (plasmids, insertion sequences, intact prophages, and integrons)

Plasmid analysis revealed that all but one isolate harboured plasmid replicons. Nineteen different replicons were found in the 23 isolates. InCFIB was the most frequently found replicon and was common to 11 isolates from patients and six from environment. The IncFIA, IncFIB, IncFII plasmid replicons were found together in twelve isolates: eight in patient isolates and four in environmental isolates. Two isolates from patients and one from the environment harboured up to seven different replicons.

The CTX-M-15 gene in both patients and environmental isolates was consistently associated with an insertion sequence of the IS380 family and a transposon, commonly Tn3, while the *bla*_TEM_ gene was carried mainly by transposons. The disinfectant resistant gene, *qacE*, which was found in six isolates from patients (*n* = 5) and environmental isolates (*n* = 1), was co-carried with *sul1* usually on a class 1 integron in all the isolates. Isolate P128, was found to have CTX-M-15 genes flanked by several transposons, insertion sequences and a recombinase and also harboured TEM-1, *qnrS1*, *aph(6)-Id*:*aph(3’’)-Ib* and *sul2.* The contig harbouring the CTX-M-15 gene in P128 showed high similarity of 99.98% to the *E. coli* strain PGR46 plasmid pPGRT46 (KM023153.1) The *bla*_OXA−1_ gene which was commonly flanked by the chloramphenicol resistant gene, *catB-3* and the aminoglycoside hydrolyzing gene, *aac(6’)-Ib-cr5* was not associated with transposons or insertion sequences. However, the contig harbouring the *bla*_OXA−1_ gene showed, showed high similarity to a plasmid, pBL12EC-2 (Supplementary table S5).

Investigation of MGEs which mobilize and transfer resistance genes between isolates revealed the presence of diverse MGEs in the isolates.

Many of the contigs of the *E. coli* isolates harbouring ARGs showed high similarity (98-100%) to plasmids in GenBank, confirming that most of the ARGs from both patients and environment are mobilized and disseminated by plasmids (Supplementary table S5).

Since prophages are known to be associated with pathogenicity factors, the types of prophages in the isolates were investigated. Twenty-five different intact prophages were identified among nineteen MDR *E. coli* isolates from patient and environments. The most common prophages were Entero_mEp460 which was identified in seven isolates (Two from environment and five from patients) and Entero_BP_4795 (five patient isolates) and Klebsi_4LV2017 (three isolates: two patients and one environmental isolate). P51B from patient and E53 from environment with ST648 had the same phages (Escher_TL_2011b and Klebsi_4LV2017) Supplementary table S6).

The most common integrons were of the class I type identified in sixteen isolates: nine isolates from patients and six from the environment. Similar integrons bearing the same gene cassettes were identified in isolates from both patients and the environment. The integrons commonly encoded genes for sulphamethoxazole, trimethoprim and aminoglycoside resistance. In54 and In191 were the most identified class I integron types, occurring in five and four isolates respectively. Gene cassettes of In54 frequently consisted of *AadA5*, *dfrA17*, *qacE* and *sul1.* The gene cassettes were common to isolates from four patients and an isolate from the environment. One other isolate from the environment (E50-1) with the In54 differed and lacked the *qacE* and *sul1* genes. The class I integron, In191 which was found with *dfrA14* occurred in four isolates from patients and one from the environment (Table 2).

Several different insertion sequences were detected in the *E. coli* isolates from patients and environments. Twenty isolates harboured at least one insertion sequence element. IS621 was the most commonly occurring insertion element and was found in thirteen isolates (Nine isolates from patients and four from the environment). The insertion sequence MITEEc1 was found in eight (six from patients and two from environments) isolates (Supplementary Table S6).

**Table 2:** Integrons, gene cassettes (GCs) and sequence types found in the *Escherichia coli* isolates

ID	SOURCE	MLST	Integron	Integron	GC1	GC2	GC3	GC4	transposon
P2R	Patient	ST940	Intl2	In2-3	*dfrA1*	*sat2*	-	-	IS256, Tn7, TnsD
P73	Patient	ST940	Intl1	In757	*oxa-1*	*AadA1*	-	-	Tn3
P143	Patient	Unknown	Intl1	In757	*oxa-1*	*AadA1*	-	-	Tn3
P128	Patient	ST13846	Intl1	In191	*dfrA14*	*-*	-	-	
P166	Patient	ST13846	Intl1	In191	*dfrA14*	*-*	-	-	
P60R	Patient	ST1722	Intl1	In191	*dfrA14*	*-*	-	-	
E37	Environment	ST410	Intl1	In191	*dfrA14*	*-*	-	-	IS6, Tn3
P142	Patient	ST5614	Intl1	In54	*AadA5*	*dfrA17*	*qacE*	*sul1*	Tn3, IS6
P159	Patient	ST617	Intl1	In54	*AadA5*	*dfrA17*	*qacE*	*sul1*	
P105	Patient	ST10	Intl1	In54	*AadA5*	*dfrA17*	*qacE*	*sul1*	
E50-1	Environment	ST155	Intl1	In54	*AadA5*	*dfrA17*	*-*	*-*	Tn3
E55-2	Environment	ST58	Intl1	In54	*AadA5*	*dfrA17*	*qacE*	*sul1*	IS6
P115	Patient	Unknown	Intl2	In2-3	*dfrA1*	*sat2*	*-*	*-*	
			Intl1	In54	*AadA5*	*dfrA17*	*-*	*-*	
E29	Environment	ST450	Intl1	In987	*dfrA17*	*-*	*-*	*-*	
E25B	Environment	ST127	Intl1	In22	*dfrA7*	*qacE*	*sul1*	*-*	Tn3

### Virulome, serotypes and phylogroups

Isolates from both patients and environments were found to harbour several virulence genes which did not differ much between sources. The most frequently identified virulence genes included *terC* which was found in 21 isolates: fourteen from patients and 7 from environment. The virulence gene *ipfA* was also harboured by 14 isolates – nine from patients and five from environment. Other frequently identified virulence genes in the isolates included, *gad* (13 isolates), *sat* (2 isolates), *papA* (7), *irp2* (11), *fyuA* (11), *iutA* (7), *traT* (12), *iucC* (7), *iss* (9), *yfcv* (6), *chuA* (6), *ompT* (7). The highest number of virulence genes were found in an isolate P49 from a patient (33 genes) which was acquired on admission. E25B, an isolate from the environment also harboured 25 virulence genes (Table 1).

The somatic (O) and flagellar (H) antigens were used for serotyping the *E. coli* isolates where twelve different O antigens and 17 different H types were identified across all isolates. No O type was detected for two isolates P73, from patient and E50-1, from environment which only had the H5 and H40 antigens respectively. The O antigen, O101 was common to four isolates (P2R, P105, P159 and P63) from patients. P105 (ST10) and P159 (ST617) had in common, the O101 and H10 antigens. Isolates P128 and P166 both of the novel ST 13,846 had the O15 antigen but varied in the H antigen (Supplementary Table S4). The complexity and diversity of the virulome in isolates from patients and from environments coupled with the range of identified capsule types is a concern for IPC as they are associated with virulence.

Isolates were found to belong to six phylogroups; with six isolates (four carriage isolates and two from environments) belonging to A and to B1. Three isolates (two from patients and one from environments) belonged to the phylogroup F, two isolates each from patients belonged to D and E, while one from a patient and one from environment belonged to the frequently virulent phylogroup B2 and E (Table 1). All phylogroups of patient and environmental isolates harboured several virulence genes.

### Sequence types and phylogenomic relationships

**Fig. 1:**
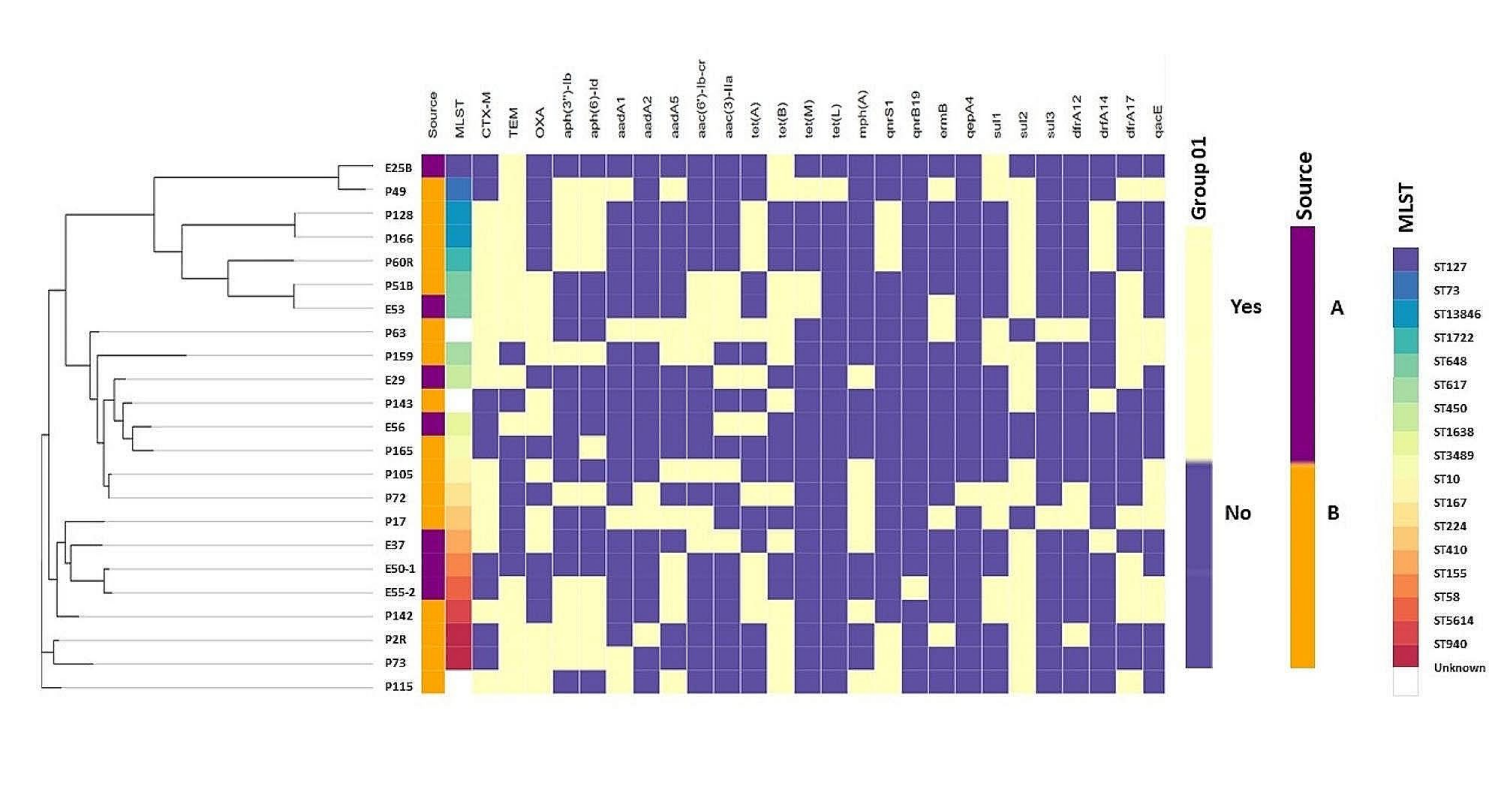
The phylogenetic branch and metadata [demographics, molecular typing, and antibiotic resistance genes (ARGs)] coupled by the use of Phandango (https://github.com/jameshadfield/phandango/wiki) in multidrug resistant *Escherichia coli* isolates (*n* = 23) from hospital patients and environments in a Teaching Hospital in Ghana

MLST analysis showed that the isolates belonged to seventeen different sequence types (ST127, ST73, ST13846, ST1722, ST648, ST617, ST450, ST1638, ST3489, ST10, ST167, ST224, ST410, ST155, ST58, ST5614, ST940). Three isolates (P143, P115 and P63) belonged to unknown STs (Table 1). ST10, ST167, ST225, ST3489, ST617, ST940, ST 13,846 were of community carriage isolates. Two isolates (P73 and P2R) from two patients had the same ST, ST 940. P73 was acquired by a patient on admission. One isolate (P51B) acquired by a patient on admission at the Obstetrics and Gynaecology directorate and one from the environment (E53) of the surgery unit had the same ST 648. Two isolates (P128 and P166) with unknown STs, both isolated from patients in different directorates after 48 h of admission were assigned a putatively novel ST13846. These isolates with same ST showed significant clonal relatedness in the phylogenetic tree (Fig. 1) and had similar resistance genes, virulence genes and plasmids (Table 1). Analyses of the genetic environment of this new ST showed the *bla*_CTX−M−15_ and *bla*_TEM−1_ genes were associated with Tn3::CTX-M-15:recombinase: TEM-1:IS91(transposase) on a contig which bore close resemblance with the *E. coli* strain PGR46 plasmid pPGRT46 (KM023153.1). This contig also carried the quinolone resistant gene *qnrS1* and the *aph(6)-id: aph(3’’)-ib: sul2* genes close to the insertion sequence IS5075 and the Tn3 transposon. P128 and P166 which shared similar resistance genes and genetic environment as P60R (ST1722), differed in the plasmid replicons. P60R, belonging to ST1722 isolated from a patient on admission, with similar resistance genes as P128 and P166 (ST13846) had in addition to the IncY plasmid, IncB/O/K/Z, IncI2(Delta) and pXuzhou21.

Generally, isolates clustered into three main groups with one other grouping of two isolates and an outlier isolate. Group A constituted seven isolates; two isolates (E25B and E53) belonging to ST127 and ST648 from environment and five isolates (P49, P128, P166, P60R and P51B) of STs ST73, ST13846, ST1722 and ST648 from patients. Eight isolates grouped into B and included two isolates (E29 and E56) belonging to ST450 and an unknown ST from environment and six isolates (P63, P143, P159, P165, P105 and P72) belonging to two unknown STs, ST617, ST3489, ST10 and ST167. The third group, C comprised of two isolates (P17 and P142) of STs 224 and ST5617 from patients and three isolates (E37, E50-1 and E55-2) which belonged to ST410, ST155 and ST48 from environments. P2R and P73 both from patients and belonging to ST940 clustered in one group while P115 (unknown ST) was an outlier isolate (Fig. 1).

**Fig. 2:**
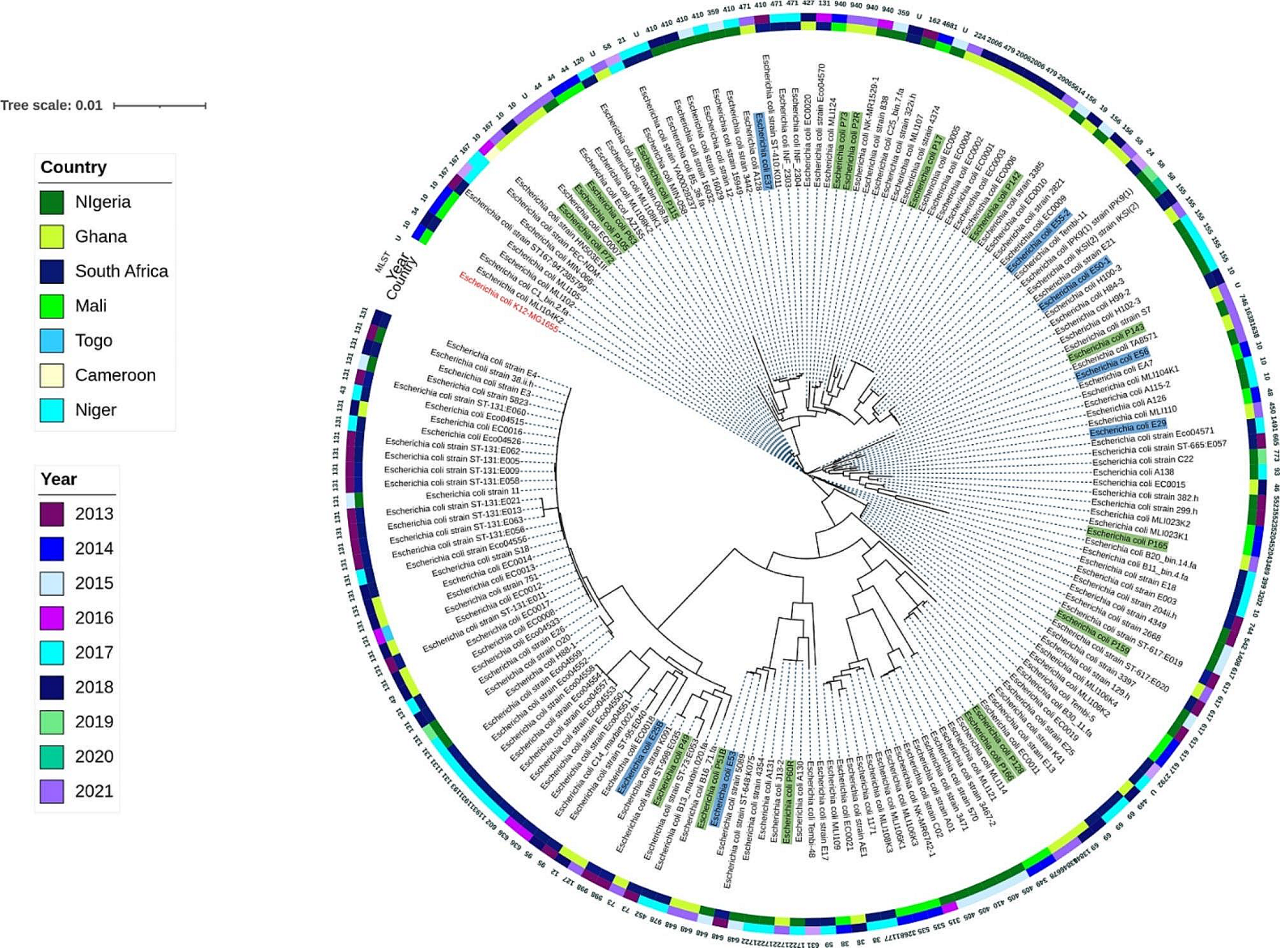
Maximum likelihood phylogenetic tree of *Escherichia coli* strains isolated from humans between 2013 and 2022 in selected African countries. The core-genome phylogenetic tree was drawn from 181 genomes with BV-BRC and annotated with iTOL. The tree was built with *E. coli* K12-MG1655 as the reference genome and rooted with the reference strain. The following metadata are indicated: the country of isolation on the inner colored ring, the year of isolation in the middle and the MLST in (isolates with unknown STs are indicated U) on the outer ring. Isolates from this study are labelled with green background (isolates from patients) and blue background (isolates from environment)

To visualize the genetic relatedness among *E. coli* isolates, we inferred a maximum likelihood phylogenetic tree from an alignment of 181 genomes including the complete genome of *E. coli* K12-MG1655 (reference genome) (Fig. 2). Isolates from this study clustered together with genomes from other countries, frequently with isolates from Nigeria and South Africa. They clustered mainly based on sequence type (Fig. 2). Isolates from the environment, clustered together with isolates of similar sequence types or variants of the sequence types from clinical sources. For instance, E50-1 (ST155), an isolate from the environment clustered together with other isolates of similar ST from clinical sources in other countries (Fig. 2).

## Discussion

This study showed that patients were colonized with genetically diverse ESBL *E. coli* isolates on admission and during hospital stay. ESBL- producing *E. coli* strains were also found to be potentially disseminated between patients and their immediate environment.

The carriage of ESBL *E. coli* isolates by patients on admission in the hospital is suggestive of community carriage and subsequent introduction of these isolates into the directorates of the hospital on admission. Higher carriage rates of ESBL *E. coli* have been observed in previous studies in Tanzania (23.7%) and Iran (60.0%) [12, 16] and lower rate (10.5%) in Israel [17]. The incidence of ESBL *E. coli* acquisition among patients who previously did not harbor ESBL *E. coli* was 11.32%, which is lower than that reported in Israel [17]. Differences in IPC practices and differences in screening time points for ESBL *E. coli* acquisition in various settings may account for the differences in carriage rates observed. Continuous screening for ESBL *E. coli* over the course of patients’ stay may contribute to the detection of acquisition events.

Molecular characterization of the MDR *E. coli* isolated revealed a diversity of resistant genes in both patient carriage and environmental isolates. Among these, the ESBL gene, CTX-M was found predominantly present in the isolates, most likely borne on plasmids and commonly found together with the insertion sequence, IS1380 and the transposase Tn3 which could disseminate this gene between isolates from different sources. The predominance of the *bla*_CTX−M 15_ gene has been reported globally and in Ghana, among ESBL *E. coli* isolates associated with infections of both community and hospital origins [18–20]. A study in Libya of clinical *E. coli* isolates reported a lower rate of 17.3% of isolates carrying the *bla*_CTX−M−15_ gene [21] compared to the 52.2% of isolates with the *bla*_CTX−M−15_ genes from this study. Though the *bla*_CTX−M_ gene is frequently associated with the globally disseminated high-risk ST ST131 [22, 23], we found the gene present in *E. coli* strains with various ST types indicating the successful transfer of the *bla*_CTX−M_ gene across various strains of *E. coli*.

There was also marked resistance to ciprofloxacin mediated by chromosomal point mutations in the *parCE* and *gyrAB* genes among isolates harbouring the *bla*_CTX−M_ gene. Fluoroquinolone resistance has often been reported in ESBL-producing *E. coli* isolates in association with the CTX-M genes [24] raising concerns about the circulation of highly resistant clones. In accordance with other studies, the class 1 integron was present in isolates, together with the CTX-M-15 genes and was found to be associated with genes encoding resistance to aminoglycosides (*aadA* gene cassettes) and/or trimethoprim (*dfrA* gene cassettes) [25, 26]. Gene cassettes with sulphonamides (*sul1*) and quaternary ammonium compounds (*qacE*) genes) were also found on class 1 integrons that were closely associated with the heavy metal resistance gene, chromium (*chrA*) resistance gene. The presence of class 1 integrons in both carriage isolates and isolates from environments could facilitate the transfer of resistance genes within and between isolates of different sources as they are noted to disseminate resistance due to their inherent mobility and are important in the development of multidrug resistance in *E. coli* [27]. Isolates co-bearing the CTX-M-15, *qacE* and *chrA* genes in both patient and environmental isolates could co-select for disinfectants, heavy metals and antibiotics [28], becoming problematic in disinfection efforts in IPCs as they are frequently associated with and mobilized by mobile genetic elements, promoting their dissemination.

An interesting finding was the presence of the carbapenemase *bla*_OXA−181_ gene in two *E. coli* isolates of ST940 from patients in the same ward, one of which did not show phenotypic resistance to the carbapenems. Notably, one isolate P73 bearing the *bla*_OXA−181_ was acquired on admission by a patient admitted nearly a month after the other patient. The *bla*_OXA−181_ gene has been reported among *E. coli* isolates from colonized patients, but usually among carbapenem resistant isolates, as reported in studies in Kuwait and Korea [29, 30] and recently in *E. coli* isolates of ST410 and ST940 from paediatric patients with diarrhoea in Ghana [31]]. The contigs that contained the *bla*_OXA−181_ gene in both isolates closely resembled the p010_B-OXA181 (CP048332.1) and pEC213_1-OXA-181(CP061102.1) plasmids suggesting a dissemination of an evolving phenotype of the *bla*_OXA−181_ gene by same plasmids as reported by Prah et al. [31] in the hospital and in Ghana.

The IncF plasmid was frequently found to be associated with resistant genes in all ST types. Acquired resistance genes in *E. coli* isolates have been found to be carried by the F plasmids and usually with transposable elements, making the mobilization and dissemination of resistant genes between isolates from various sources possible [32].

The detection of a high number of similar virulence genes in the *E. coli* isolates from both patient carriage and environment suggests a high pathogenicity of isolates from both community and hospital environments (Table 1). Virulence factors such as the adhesins *iha*,* papA*,* papC*, *sfaD*, *sfaE* and *sfaS* for adherence and invasion, as well as toxins *hylA* and *hylE* on virulence genes harboured by the isolates from both patients and environments in this study enable pathogens to survive for longer periods in colonized hosts thereby increasing their chances of eventually causing disease [33]. Coupled with the presence of several virulence genes in the phylogroups A and B1 which were dominant in patient carriage and environmental contamination isolates, there is the potential for increased risk of infection in patients just as isolates of the phylogroups B2 and D which are notably extra-intestinal and virulent [34, 35].

The isolates were found to belong to different STs, of which some were found common to some isolates. Two isolates, from a patient and environment of the same sequence type, ST648 carried similar antibiotic resistance genes, particularly the β-lactamases, *bla*_CTX−M_, *bla*_TEM_ and *bla*_OXA−1_ genes (Table 1). These two isolates were also 100% phylogenetically related and interestingly isolated within the same month, from different directorates, i.e. from a patient in the Obstetrics and Gynaecology ward and from a bed in the ICU (Supplementary table S1). The patient had acquired this isolate on admission, suggesting an inter-ward dissemination of the same strain spreading between patients and environment. ST648 *E. coli* strains, with their biofilm adherence virulence genes have emerged globally as a highly virulent strain likely to cause bacteremia just like the internationally circulating ST131 strain [36] which is first reported among human isolates from Ghana in this study.

Similarly, two isolates from two patients in the Obstetrics and Gynaecology and the Surgical directorates belonged to ST940, one of which was acquired on admission a month after isolation of the other isolate which was carried by a patient into the ward. A novel sequence type ST13846 of the virulent phylogroup D which was detected in two isolates showed high clonal relatedness on phylogenetic analyses. Notably, these isolates were obtained from rectal swabs of two patients in two different directorates, i.e. Obstetrics and Gynaecology and Surgery within the same month and possibly acquired on hospitalization, suggesting a rapid dissemination of this new ST within the hospital. The resistance genes of this new sequence type appeared to be associated with several MGEs which could facilitate the rapid spread of this the antibiotic resistance genes carried by isolates of the new ST within patients in the hospital.

The detection of isolates of common sequence types to patients and in some instances, environment in this study suggests an introduction and subsequent circulation of the ESBL *E. coli* isolates in the hospital.

Other high-risk STs detected among isolates from patients or environment in this study included ST410, ST1722 and ST10, which have also been observed among *E. coli* isolates from Ghana [15, 19, 37–39]. Phylogenetic analyses of the isolates revealed the clustering of *E. coli* isolates from patients and environments and in different directorates into similar groups (Fig. 1), suggesting an inter-ward spread of the strains. The clustering of isolates from this study belonging to the high-risk sequence types ST131, ST617, ST648 among others with clinical isolates of same sequence types from other African countries reveals the endemicity of these STs in the Africa. Indeed, these STs have been reported among clinical isolates from infections in Nigeria, Ethiopia and South Africa [40–42]. The isolate with novel ST13846 clustered together with strains of ST69, indicating a likelihood of the novel ST being a variant of the ST69 which may have been imported from other African countries. The clustering of isolates colonizing patients and contaminating environments with high-risk clones of clinical isolates from other countries suggest the potential of the isolates from this study to cause clinical infections in patients in the absence of efficient IPCs. There is the need for continuous surveillance for the timely detection of imported antibiotic resistant strains. The isolates were closely related mainly with isolates from Nigeria and South Africa noting that these countries have uploaded the largest number of genomes on the open access databases.

Since rectal screening for ESBLs is not a practice in the hospital, patients who did not consent to screening or did not meet the inclusion criteria could have been missed out as potential sources of acquisition. Environmental surfaces which were not sampled could also have harboured the isolates which were acquired.

This study has revealed the possible dissemination of MDR ESBL-producing *E. coli* isolates in the hospital between patients and environments, highlighting the necessity of screening patients for carriage of these pathogenic organisms.

This study has also highlighted the importance of using genomic data to detect isolates in patient carriage and environments which may have associations with strains that can cause infections.

## Conclusion

MDR ESBL *E. coli* isolates were found to be circulating among hospitalized patients and their environment in Ghana. These isolates had a repertoire of resistance and virulence genes mainly associated with plasmids making them highly mobile. This is a concern for strengthening IPC practices and surveillance of not only isolates from patients but also from the environment in order to prevent and contain the spread of MDR organisms in hospitals in Ghana.

### Electronic supplementary material

Below is the link to the electronic supplementary material.

Supplementary Material 1

## Data Availability

All contiguous sequences have been submitted to GenBank and assigned accession numbers under BioProject PRJNA823741. Other datasets used and/or analysed during the current study are available from the corresponding author on reasonable request.
