# Supplementary material for: Genomic characterization of multi drug resistant ESBL-producing Escherichia coli isolates from patients and patient environments in a teaching hospital in Ghana
Source: BMC Microbiol. 2024 Jul 8;24:250. doi: 10.1186/s12866-024-03406-1 (PMC11229298; doi:10.1186/s12866-024-03406-1)
Supplement: Supplementary file 1 — Supplementary Material 1 [file 12866_2024_3406_MOESM1_ESM.docx]

**Supplementary tables**

**Table S1: Characteristics of E. coli isolates from patients and environment**

| **Bacterial ID** | **Patient no.** | **Date of collection** | **Source** | **Directorate** | **Source** | **Sample** |
| --- | --- | --- | --- | --- | --- | --- |
| P105 | AP6 | 1/6/2021 | Patient | Surgery | Rectal | admission |
| P128 | AP63 | 22/7/2021 | Patient | Obstetrics & gynaecology | Rectal | after 48h |
| P72 | AP40 | 18/5/2021 | Patient | Obstetrics & gynaecology | Rectal | admission |
| P60R | AP66 | 30/7/2021 | Patient | Obstetrics & gynaecology | Rectal | Admission* |
| P17 | BP17 | 30/04/2021 | Patient | Obstetrics & gynaecology | Rectal | admission |
| P165 | AP68 | 27/7/2021 | Patient | Surgery | Rectal | admission |
| P142 | AP67 | 27/7/2021 | Patient | Obstetrics & gynaecology | Hand | admission |
| P159 | AP13 | 30/7/2021 | Patient | Obstetrics & gynaecology | Rectal | admission |
| P51B | AP32 | 15/5/2021 | Patient | obstetrics & gynaecology | Rectal | after 48h |
| P49 | AP31 | 15/5/2021 | Patient | Obstetrics & gynaecology | Rectal | after 48h |
| P2R | AP2 | 30/4/2021 | Patient | Obstetrics & gynaecology | Rectal | admission |
| P73 | AP40 | 21/5/2021 | Patient | Obstetrics & gynaecology | Rectal | after 48h |
| P166 | BP13 | 30/7/2021 | Patient | Surgery | Rectal | After 48h |
| P143 | AP68 | 27/7/2021 | Patient | Obstetrics & gynaecology | Rectal | admission |
| P115 | AP56 | 15/6/2021 | Patient | Obstetrics & gynaecology | Rectal | admission |
| P63 | AP29 | 12/5/2021 | Patient | Obstetrics & gynaecology | Rectal | admission |
| E25B | bed | 21/5/2021 | Environment | Obstetrics & gynaecology | Bed |  |
| E50-1 | dripstand | 30/4/2021 | Environment | Obstetrics & gynaecology | dripstand |  |
| E56 | bed | 3/5/2021 | Environment | Surgery | bed |  |
| E37 | tap | 19/7/2021 | Environment | Obstetrics & gynaecology | tap |  |
| E29 | tap | 14/6/2021 | Environment | Obstetrics & gynaecology | tap |  |
| E55-2 | bed | 1/6/2021 | Environment | Surgery | bed |  |
| E53 | bed | 3/5/2021 | Environment | Surgery/ ICU | bed |  |

**Table S2: Genome and assembly characteristics of sequenced *E. coli* isolates from patients and environment**

| **ISOLATE ID** | **SOURCE** | **ACCESION NO.** | **Antigen**  **(Somatic O)** | **Flagellar (H)** | **SEQUENCE LENGTH** | **NO. OF CONTIGS** | **GC CONTENT %** | **Longest contig size (bp)** | **N50 value** | **L50 value** |
| --- | --- | --- | --- | --- | --- | --- | --- | --- | --- | --- |
| P51B | Patient | SAMN27356662 | O102, O102 | H6 | 5199290 | 136 | 50.5 | 693002 | 162647 | 10 |
| P2R | Patient | SAMN27356667 | O101 | H4, H21 | 4734033 | 141 | 50.7 | 338266 | 107713 | 14 |
| P49 | Patient | SAMN27356668 | O6 | H1 | 5976865 | 3186 | 50.2 | 40717 | 2786 | 488 |
| P73 | Patient | SAMN27356669 | No hit | H5 | 4793808 | 157 | 50.7 | 261280 | 93708 | 16 |
| P142 | Patient | SAMN27356671 | O27 | H14 | 5763277 | 956 | 50.7 | 76312 | 15233 | 112 |
| P17 | Patient | SAMN27356672 | O8 | H23 | 5081498 | 137 | 50.8 | 568303 | 206541 | 9 |
| P60R | Patient | SAMN27356674 | O1, O1 | H25 | 5144797 | 78 | 50.4 | 551539 | 219648 | 7 |
| P165 | Patient | SAMN27356676 | O61, O61 | H34 | 4780747 | 57 | 50.6 | 646885 | 189779 | 8 |
| P105 | Patient | SAMN27356677 | O101, O101 | H10 | 5067872 | 181 | 50.5 | 187302 | 89215 | 19 |
| P159 | Patient | SAMN27356678 | O101, O101 | H10 | 4932348 | 134 | 50.7 | 221547 | 106189 | 17 |
| P166 | Patient | SAMN27356679 | O15, O15 | H10 | 5803192 | 1590 | 50.6 | 72453 | 13132 | 119 |
| P128 | Patient | SAMN27356680 | O15, O15 | H18 | 4967815 | 85 | 50.6 | 423144 | 186239 | 9 |
| P115 | Patient | SAMN27356683 | O7, O53 | H4 | 5983896 | 3109 | 50.2 | 252708 | 3104 | 451 |
| P143 | Patient | SAMN27356684 | O99, O99, O8 | H20 | 5657046 | 3098 | 50.5 | 58301 | 2839 | 465 |
| P63 | Patient | SAMN27356685 | O101, O101 | H4 | 6187348 | 3779 | 50.6 | 60134 | 2496 | 605 |
| P72 | Patient | SAMN31146634 |  |  | 4711974 | 124 | 50.8 | 254392 | 118922 | 14 |
| E29 | Environment | SAMN27356664 | O45, O45 | H19 | 4981043 | 183 | 50.7 | 180939 | 86806 | 20 |
| E25B | Environment | SAMN27356665 | O6 | H31 | 5206655 | 134 | 50.4 | 423782 | 132761 | 14 |
| E53 | Environment | SAMN27356666 | O102, O102 | H6 | 5188919 | 152 | 50.5 | 530199 | 137528 | 12 |
| E50-1 | Environment | SAMN27356673 | no hit | H40 | 4659426 | 88 | 50.6 | 387639 | 118999 | 13 |
| E55-2 | Environment | SAMN27356675 | O8 | H30 | 4947644 | 145 | 50.8 | 288469 | 105077 | 16 |
| E56 | Environment | SAMN27356681 | O53 | H18 | 5164605 | 861 | 50.1 | 208569 | 37344 | 44 |

**Table S3:** Chromosomal point mutations of gyrA/B, parC/E genes of the *E. coli* isolates

| **ISOLATE ID** | **gyrA** | **gyrB** | **parC** | **parE** |
| --- | --- | --- | --- | --- |
| P51B | S83L, D87N, *A828S, *D678E | S492N, A618T, E656D | E62K, S801, D475E | T172A, S458A |
| P49 | S83L |  |  |  |
| P17 | S83L, D87N |  | S80I | S458A |
| P60R | *A863V | *S492N, *A618T | *E62K, *D475E |  |
| P105 | S83L, D87N |  | S80I | S458A |
| P159 | S83L, D87N |  | S80I | S458A |
| P128 | *D678E | *E703D | *E62K |  |
| P166 |  | *E703D | *E62K |  |
| P143 | D678E |  | E62K |  |
| P63 | S83L, D87N |  | E62K, S801, L440R | S458A |
| P72 | S83L, D87N |  | E62K, S801 | S458A |
| E29 | S83L, D87N |  | S80I | S458A |
| E25B | S83L, D87N |  | S80I | S458A |
| E53 | S83L, D87N |  | S80I | S458A |
| E50-1 |  |  | E62K |  |
| E56 |  |  | E62K |  |
| E37 | S83L, D87N |  | S80I | S458A |

**Putatively novel mutations*

**Table S4: Capsular types of *E. coli* isolates**

| **ISOLATE ID** | **SOURCE** | **MLST** | **Antigen**  **(Somatic O)** | **Flagellar (H)** |
| --- | --- | --- | --- | --- |
| P51B | Patient | ST648 | O102, O102 | H6 |
| P2R | Patient | ST940 | O101 | H4, H21 |
| P49 | Patient | ST73 | O6 | H1 |
| P73 | Patient | ST940 | No hit | H5 |
| P142 | Patient | ST5614 | O27 | H14 |
| P17 | Patient | ST224 | O8 | H23 |
| P60R | Patient | ST1722 | O1, O1 | H25 |
| P165 | Patient | ST3489 | O61, O61 | H34 |
| P105 | Patient | ST10 | O101, O101 | H10 |
| P159 | Patient | ST617 | O101, O101 | H10 |
| P166 | Patient | ST13846 | O15, O15 | H10 |
| P128 | Patient | ST13846 | O15, O15 | H18 |
| P115 | Patient | Unknown | O7, O53 | H4 |
| P143 | Patient | Unknown | O99, O99, O8 | H20 |
| P63 | Patient | Unknown | O101, O101 | H4 |
| P72 | Patient | ST167 | no hit | no hit |
| E37 | Environment | ST410 | O8, O8 | H9 |
| E29 | Environment | ST450 | O45, O45 | H19 |
| E25B | Environment | ST127 | O6 | H31 |
| E53 | Environment | ST648 | O102, O102 | H6 |
| E50-1 | Environment | ST155 | no hit | H40 |
| E55-2 | Environment | ST58 | O8 | H30 |
| E56 | Environment | Unknown | O53 | H18 |

**Table S5: Mobile genetic elements associated with antibiotic resistance**

| **ID(ST)** | **Source** | **contig** | **Synteny of resistant genes** | **Plasmid/chromosomal sequence with closest nucleotide homology** |
| --- | --- | --- | --- | --- |
| P51B (ST648) | Patient | 113 | IS1:dfrA17: | *E. coli*702/18 plasmid p702_18_2 (CP074703.1) |
|  |  | 83 | catB3:oxa-1:AAC(6')-Ib-cr5 | *E. coli* JNQH498 plasmid pJNQH498-1 (CP104385.1) |
|  |  | 59 | aac(3)-Iid:IS4 | *E. coli* 702/18 plasmid p702_18_2 (CP074703.1) |
|  |  | 81 | IS3::aac(3)-IIe | *Enterobacter hormaechei* strain 80014967 plasmid pE80014967-1 (CP104468.1) |
|  |  | 67 | transposase::::sul2::repA | *Klebsiella quasipneumoniae* strain SWHEFF_72 plasmid unnamed1 (CP055011.1) |
|  |  | 71 | IS1:tetC:tetB:tetR(B):: | *E. coli* D16EC0456 plasmid pD16EC0456-1 (CP088600.1) |
|  |  | 64 | IS1380(transposase):CTX-M-15::Tn3 | *Klebsiella pneumoniae* strain 2021CK-01815 plasmid unnamed1 (CP104374.1) |
|  |  | 75 | erm(B)::transposase: | *E. coli* YJ6 plasmid pYJ6-NDM5 DNA (AP023236.1) |
|  |  | 69 | IS6:mpR(A):mrxA:mphA | *E. coli* plasmid pM105_mF DNA (AP018137.1) |
| P2R(ST940) | Patient | 4 | IntI2(integrase):dfrA1:sat2::IS256(transposase):Tn7(transposase):TnsD(transposase): | *E. coli* Z0117EC0040 chromosome (CP098211.1) |
|  |  | 66 | IS6(transposase):transposase::oxa-181:::ISKra4(transposase):recombinase::recombinase:QnrS1:transposase | *E. coli* 10 plasmid p010_B-OXA181 (CP048332.1) |
|  |  | 84 | IS91(transposase):APH(6)-Id:aph(3'')-Ib:sul2 | *E. coli* 1EC213 plasmid pEC213_1-OXA-181 (CP061102.1) |
|  |  | 88 | TetC:TetB:tetR(B): | *E. coli* PI24 plasmid pYLPI24a, complete sequence (CP074013.1) |
| P49(ST73) | Patient | 1123 | AadA5:dfrA17 | *Shigella flexneri* 2a strain 18787_5_65 plasmid pKSR100 (CP090162.1) |
|  |  | 127 | sul1:qacE:aadA1:dfrA1 | *E. coli* EcPF5 plasmid p1 (CP054237.1) |
|  |  | 144 | tetR(B):TetB:tetC::IS4(transposase) | *E. coli* plasmid p2 (MT077881.1) |
|  |  | 503 | sul2::helicase | *Citrobacter youngae* strain CF10 plasmid pCF10-tmexCD1 (CP102501.1) |
|  |  | 1083 | APH(6)-Id:aph(3'')-Ib: | *E. coli* THB42-F3 plasmid pHB42-F3 (CP104331.1) |
|  |  | 1131 | TEM-1:recombinase | *Salmonella enterica* strain s12177 plasmid ps12177-CTX (CP101349.1) |
| P73 (ST940) | Patient | 67 | transposase:Tn3(transposase)::oxa-181:EreA::ISKra4(transposase):recombinase::recombinase:QnrS1:transposase | *E. coli* 10 plasmid p010_B-OXA181 (CP048332.1) |
|  |  | 76 | aadA1:oxa-1:intIL(integrase)::recombinase:Tn3(transposase):catA | *Salmonella enterica* subsp. *enterica serovar* Wien strain ZM3 plasmid pZM3 (MK797990.1) |
|  |  | 79 | recombinase:TEM-190:::IS91(transposase):aph(3'')-Ib:sul2 | *E. coli* 1EC213 plasmid pEC213_1-OXA-181 (CP061102.1) |
|  |  | 83 | tetR(B):tetB:tetC::IS4(transposase) | *E. coli* plasmid p2 (MT077881.1) |
| P142(ST5614) | Patient | 86 | tetA:tetR(A):relaxase:Tn3(transposase):recombinase::IntIL(integrase):dfrA17:AadA5:QacE:sul1:chrA:::IS6(transposase):MphR(A) | *E. coli* H8 plasmid B (CP010174.1) |
|  |  | 122 | ISKra4(transposase):recombinase:QnrS1:IS3(transposase):Tn3(transposase)::CTX-M-15::: | *E. coli* O169:H41 strain 2014EL-1345-2 plasmid unnamed3(CP024226.1) |
|  |  | 151 | sul2::IS91(transposase)::APH(6)-Id:aph(3'')-Ib: | *E. coli* PMV-1 pHUSEC411like plasmid (HG428756.1) |
|  |  | 258 | tetC:tetB:tetR(B): | *E. coli* 542093 plasmid p542093_2, (CP091411.1) |
| P17(ST224) | Patient | 80 | catB:oxa-1:AAC(6')-Ib-cr5 | *E. coli* JNQH498 plasmid pJNQH498-1 (CP104385.1) |
|  |  | 69 | sul1:QacE:AadA5:dfrA17: | *E. coli* TREC4 plasmid pTREC4 (MN158990.1) |
|  |  | 48 | sul3::IS256(transposase):QacL:aadA1:cmlA1:aadA2:DfrA12 | *E. coli* A241 plasmid pA241-TEM (MN807689.1) |
|  |  | 62 | Erm(B)::transposase: | *E. coli* YJ6 plasmid pYJ6-NDM5 (AP023236.1) |
|  |  | 65 | tet(A):tetR(A):relaxase | *E. coli* E1 plasmid p3 (CP104506.1) |
| P60R  (ST1722) | Patient | 32 | recombinase:ISKra4(transposase):recombinase:qnrS1:IS3:Tn3::CTX-M-15:IS1380(transposase):::TEM-1:IS91(transposase):APH(6)-Id:APH(3'')-Ib:sul2:IS110:Tn3:transposase | *E. coli* PGR46 plasmid pPGRT46 (KM023153.1) |
|  |  | 60 | drfA14:intI1 | *E. coli* MB50 plasmid pYLMB50a (CP073950.1) |
|  |  | 55 | tet(A): tetR(A):relaxase | *C. sakazakii* strain Crono-589 plasmid pCrono589-1 (CP080592.1) |
| E55-2 (ST58) | Environment | 68 | IntI1(integrase):dfrA17:AadA5:QacE:sul1:chrA::IS6(transposase):mph(A):IS6(transposase) | *E. coli* SCU-103 plasmid pSCU-103-1 (CP054458.1) |
|  |  | 82 | tetA::transposase:tetR(A):relaxase:APH(6)-Id:aph(3'')-Ib:sul2: | *E. coli* SCU-103 plasmid pSCU-103-1 (CP054458.1) |
| P105(ST10) | Patient | 105 | intI1:dfrA17:AadA5:QacE:sul1:chrA:padR:: | *E. coli* AH62 plasmid pAH62-3 (CP055262.1) |
|  |  | 127 | transposase: CTX-M-15: | *Klebsiella pneumoniae* strain 2021CK-01815 plasmid unnamed1 (CP104374.1) |
|  |  | 145 | CatB3:OXA-1:AAC(6')-Ib-cr5 | *E. coli* IPCEC48 plasmid pIPCEC48_1 DNA (AP026795.1) |
|  |  | 140 | IS3::AAC(3)-Iie | *E. coli* p11B plasmid unnamed1 (CP103756.1) |
|  |  | 111 | tetB:tetC:AraC:Is4-like element(transposase) | *E. coli* plasmid p2 (MT077881.1) |
|  |  | 126 | Is6(transposase):MphR(A):mrx(A):mph(A) | *E. coli* IPCEC48 plasmid pIPCEC48_1 DNA (AP026795.1) |
|  |  | 118 | catA1::Tn3(transposase):recombinase | *E. coli* EC45 plasmid pEc45_1, (CP059126.1) |
| P159  (ST617) | Patient | 94 | catB:oxa-1:AAC(6')-Ib-cr5 | *E. coli* IPCEC48 plasmid pIPCEC48_1 DNA (AP026795.1) |
|  |  | 88 | TetR(B):TetB: | *E. coli* 1579 plasmid pMB3176_1 (CP103719.1) |
|  |  | 83 | sul1:QacE:AadA5:dfrA17:intI1: | *E. coli* AH62 plasmid pAH62-3 (CP055262.1) |
|  |  | 80 | sul2:::IS91(transposase):APH(6)-Id:aph(3'')-Ib: | *Klebsiella pneumoniae* subsp. *pneumoniae* strain WRC18_CMC307MC plasmid pCMC307M_P4 (CP079633.1) |
|  |  | 89 | ISEcp1(transposase):CTX-M-15:: | *Klebsiella pneumoniae* strain 2021CK-01815 plasmid unnamed1 (CP104374.1) |
|  |  | 87 | IS6(transposase):mphR(A):mrx(A):Mph(A) | *E. coli* IPCEC48 plasmid pIPCEC48_1 DNA (AP026795.1) |
| P166(ST13846) | Patient | 488 | dfrA14:intI1: | *E. coli* BL12 plasmid pBL12EC-2 (CP079777.1) |
|  |  | 388 | IS91:aph(3'')-Ib:sul2 | *E. coli* 32-4 plasmid p32-4_C (CP048313.1) |
|  |  | 131 | IS1380:CTX-M-15::Tn3:IS3:QnrS1:recombinase:ISKra4:recombinase::: | *E. coli* RIVM_C029494 chromosome (CP068823.1) |
|  |  | 426 | TEM-1:recombinase::IS1380 | *Klebsiella pneumoniae* strain 197 plasmid pMB2966_1 (CP103730.1) |
|  |  | 357 | tet(A):tetR(A):relaxase | *E. coli* ST12468 plasmid pMB3825A_1 (CP103695.1) |
| P128(ST13846) | Patient | 59 | dfrA14:IntI1 | *E. coli* BL12 plasmid pBL12EC-2 (CP079777.1) |
|  |  | 36 | recombinase:ISKpn19(transposase):recombinase:QnrS1:IS3 Tn3::CTX-M-15:recombinase:TEM1:IS91(transposase):APH(6)-Id:APH(3'')Ib:sul2::IS5075:Tn3: | *E. coli* PGR46 plasmid pPGRT46 (KM023153.1) |
|  |  | 51 | tet(A):TetR(A):relaxase | *E. coli* ST12468 plasmid pMB3825A_1, complete sequence (CP103695.1) |
| P115 (unknown ST) | Patient | 25 | recombinase:ISKra4(transposase):recombinase:qnrS1:IS3(transposase):Tn3(transposase)::CTX-M-15:IS1380(transposase):transposase | *E. coli* RM-055-WU chromosome (CP050210.1) |
|  |  | 103 | sat2:dfrA1:IntI2(integrase)::lpfA | *E. coli* 61 chromosome (CP048326.1) |
|  |  | 172 | catA1::Tn3(transposase):recombinase | *Klebsiella pneumoniae* strain 39427 plasmid pKPN39427.1 (CP054265.1) |
|  |  | 178 | tetR(B):tetB:tetC::IS4(transposase) | *E. coli* plasmid p2 (MT077881.1) |
|  |  | 123 | sul2::IS91(transposase)::recombinase: | *E. coli* plasmid p33 (MT077884.1) |
|  |  | 371 | transposase:mphR(A):mrx:mph(A): | *E. coli* YJ3 plasmid pYJ3-a DNA (AP023228.1) |
|  |  | 316 | TEM-190:recombinase:IS6(transposase) | *E. coli* UK_Dog_Liverpool plasmid pCARB35_02 (CP031655.1) |
|  |  | 417 | AadA5:dfrA17:intI1(integrase): | *E. coli* IPCEC31 plasmid pIPCEC31_1 DNA (AP026784.1) |
| P143 (unknown ST**)** | Patient | 63 | aadA1:oxa-1:IntI1(integrase)::recombinase:Tn3(transposase):catA1 | *E. coli* US32 chromosome (CP048606.1) |
|  |  | 104 | sul2::IS91(transposase):APH(6)-Id:APH(3'):dfrA14: | *E. coli* RHB03-C12 plasmid pRHB03-C12_3 (CP058033.1) |
|  |  | 217 | IS1(transposase)::tetC:tetB:tetR(B): | *E. coli* YJ3 plasmid pYJ3-a (AP023228.1) |
| P63 (unknown ST) | Patient | 52 | dfrA12::AadA3:CmlA:AadA1:QacL:IS256(transposase):sul3::: | *E. coli* CFSAN061769 plasmid pCFSAN061769_03 (CP042972.1) |
|  |  | 407 | sul1:QacE:AadA5:drfA17: | *E. coli* IPCEC48 plasmid pIPCEC48_1 DNA (AP026795.1) |
|  |  | 738 | catB3:oxa-1: AAC(6')-Ib-cr5 | *E. coli* JNQH498 plasmid pJNQH498-1 (CP104385.1) |
|  |  | 877 | ampR:*bla*DHA-1 | *E. coli* 142 plasmid p142_A-OXA181 (CP048338.1) |
|  |  | 517 | IS1380(transposase):CTX-M-15: | *E. coli* MS6192 chromosome (CP054940.1) |
|  |  | 769 | TEM-1:recombinase | *E. coli* TQ2 plasmid pTQ6-tet(X4) (ON390814.1) |
|  |  | 272 | tet(A):tetR(A):Tn3(transposase) | *E. coli* CP8-3_Sichuan plasmid pCP8-3-IncFIB (CP053738.1) |
|  |  | 273 | tetC:tet(B):tetR(B) | *E. coli* YJ3 plasmid pYJ3-a DNA (AP023228.1) |
|  |  | 302 | IS6(transposase):MphR(A):mrx:mphA | *E. coli* THB42-F3 plasmid pHB42-F3 (CP104331.1) |
| P72(ST167) | Patient | 69 | qacE:sul1::chrA::IS6(transposase):mphR(A): | *E. coli* Ecol_881 plasmid pEC881_1(CP019028.1) |
|  |  | 77 | IS6(transposase):eamA:tetA:tetR(A):relaxase: APH(6)-Id: aph(3'')-Ib:sul2:repC | *E. coli* HP243 plasmid pHP243 DNA(LC520289.1) |
|  |  | 94 | AadA1: DUF1010:drfA12: | *Cronobacter sakazakii* strain Crono-589 plasmid pCrono589-1(CP080592.1) |
|  |  | 75 | catA1:tn3(transposase)::recombinase: DUF3330:int1L(integron) | *E. coli* dm654b plasmid p_dm654b_NDM5(CP095637.1) |
|  |  | 85 | tetR(B):tetB:tetC:AraC:IS4(transposase) | *E. coli* 2021CK-00607 plasmid unnamed1(CP104666.1) |
|  |  | 97 | CTX-M-27: IS5/IS1182(transposase) | *E. coli* SCU-103 plasmid pSCU-103-1(CP054458.1) |
|  |  | 81 | QepA4:IS91(transposase) | *E. coli* isolate MSB1_8B-sc-2280300 genome assembly, plasmid: 2 (LR890537.1) |
| E50-1(ST155) | Environment | 72 | sul2::IS110 | *E. coli* CFS3313 plasmid pCFS3313-2 (CP026941.2) |
|  |  | 65 | AadA5:dfrA17:intI1(integrase):recombinase:Tn3(transposase) | *E. coli* isolate J31 plasmid pJ31 (CP053788.1) |
|  |  | 31 | relaxase:tetR(A):tet(A)::Tn3:::::::::TehB:ydcL:tnpA(transposase):insQ(transposase)::: | *E. coli* NCTC11129 genome assembly (LR134222.1) |
| E37 (ST410) | Environment | 55 | mphA:mphR(A):IS6:::dfrA14:IntI1:::Tn3:catA1 | *E. coli* 165 chromosome (CP020509.1) |
|  |  | 82 | catB-3:oxa-1:AAC(6')-Ib-cr5 | *E. coli* JNQH498 plasmid pJNQH498-1 (CP104385.1) |
|  |  | 70 | APH(6)-Id:sul2::: | *Salmonella enterica* subsp. *enterica* strain SCSM4.1 chromosome (CP047115.1) |
|  |  | 67 | IS1380(transposase):CTX-M-15::Tn3 | *E. coli* 1EC187 chromosome (CP061108.1) |
|  |  | 82 | catB-3:oxa-1:AAC(6')-Ib-cr5 | *E. coli* JNQH498 plasmid pJNQH498-1 (CP104385.1) |
|  |  | 73 | TetC:TetB:TetR(B)::: | *E. coli* 1EC187 plasmid pEC187_1 (CP061109.1) |
|  |  | 79 | IS3::aac(3)-Iie: | *E. coli* PM22 plasmid pYLPM22a (CP074020.1) |
| E29(ST450) | Environment | 90 | transposase:eamA:tetA:tetR(A):relaxase:APH(6)-Id: APH(3'')-Ib:sul2: | *E. coli* SCU-103 plasmid pSCU-103-1 (CP054458.1) |
|  |  | 105 | erm(B)::transposase: | *E. coli* 5M plasmid pISV_IncFII_NDM-5 (MN218686.1) |
|  |  | 92 | TEM-1:recombinase:IS6(transposase):mphR(A):mrxA:mph(A) | *E. coli* plasmid pV021-b (AP014876.1) |
|  |  | 137 | dfrA17:IntI1(integrase) | *E. coli* IPCEC31 plasmid pIPCEC31_1 DNA(AP026784.1) |
|  |  | 109 | catA1::Tn3: | *Klebsiella pneumoniae* isolate 11 genome assembly, plasmid: P1 (OX030692.1) |
|  |  | 125 | AAC(3)-Iid:IS4(transposase) | *E. coli* AH62 plasmid pAH62-3 (CP055262.1) |
| E25B(ST127) | Environment | 83 | Tet(B):TetC:IS4 | *E. coli* plasmid p2 (MT077881.1) |
|  |  | 62 | sul1:qacE:dfrA7:IntI1::recombinase:Tn3::catB3 | *E. coli* SCU-397 plasmid pSCU-397-2 (CP054830.1) |
|  |  | 86 | Tn3::TEM-1 | *E. coli* 1190 plasmid p86 (CP023387.1) |
| E53(ST648) | Environment | 69 | transposase::::sul2::helicase | *Klebsiella quasipneumoniae* strain SWHEFF_72 plasmid unnamed1 (CP055011.1) |
|  |  | 89 | catB3:oxa-1:AAC(6')-Ib-cr5: | *E. coli* strain JNQH498 plasmid pJNQH498-1 (CP104385.1) |
|  |  | 72 | IS1(transposase:TetC:TetB:TetR(B):: | *E. coli* D16EC0456 plasmid pD16EC0456-1 (CP088600.1) |
|  |  | 128 | IS1(transposase):dfrA17 | *E. coli* 702/18 plasmid p702_18_2 (CP074703.1) |
|  |  | 68 | IS1380(transposase):CTX-M-15::Tn3(transposase) | *Klebsiella pneumoniae* strain 2021CK-01815 plasmid unnamed1 (CP104374.1) |
|  |  | 61 | AAC(3)-Iid:IS4(transposase) | *E. coli* plasmid p5 DNA, strain: SA1-12-GR-1 (LC318095.1) |
|  |  | 84 | IS3(transposase)::aac(3)-Iie: | *Enterobacter hormaechei* strain 80014967 plasmid pE80014967-1 (CP104468.1) |
|  |  | 75 | ermB::transposase | *E. coli* YJ6 plasmid pYJ6-NDM5 (AP023236.1) |
|  |  | 70 | recombinase:IS6:mphR(A):mrxA:mphA | *E. coli* plasmid pM105_mF DNA (AP018137.1) |
| E56 | Environment | 195 | IS3::aac(3)-Iie | *E. coli* 100 plasmid p100_NDM5_IncN (MT199177.1) |
|  |  | 167 | tet(A):tetR(A):relaxase | *E. coli* CFS3273 plasmid pCFS3273-1 (CP026933.2) |
|  |  | 243 | catB3:oxa-1 | *E. coli* IPCEC48 plasmid pIPCEC48_1 (AP026795.1) |

**Table S6: Distribution of Insertion sequences and intact prophages among *E. coli* isolates**

| **ISOLATE**  **ID** | **SOURCE** | **MLST** | **INSERTION SEQUENCES** | **INTACT PROPHAGES** |
| --- | --- | --- | --- | --- |
| P51B | Patient | ST648 |  | PHAGE_Escher_TL_2011b_NC_019445(42), PHAGE_Klebsi_4LV2017_NC_047818(28) |
| P2R | Patient | ST940 | IS609, MITEEc1, IS621, MITEYpe1 | PHAGE_Entero_BP_4795_NC_004813(8), PHAGE_Entero_mEp460_NC_019716(19) |
| P49 | Patient | ST73 | IS621, ISChpi1, ISKol11, ISMsm1 | PHAGE_Entero_phiFL1A_NC_013646(17), PHAGE_Escher_phiV10_NC_007804(38), PHAGE_Klebsi_4LV2017_NC_047818(21), PHAGE_Entero_P88_NC_026014(22), PHAGE_Shigel_SfII_NC_021857(23) |
| P73 | Patient | ST940 | IS621, ISNisp2, ISDin1, ISAlw6 | PHAGE_Entero_BP_4795_NC_004813(7) |
| P142 | Patient | ST5614 | MITEEc1, MITEYpe1, IS621, Tn4430 | PHAGE_Entero_BP_4795_NC_004813(21) |
| P17 | Patient | ST224 | MITEEc1, MITEYpe1, IS621, Tn4430 | PHAGE_Salmon_SJ46_NC_031129(78) |
| P60R | Patient | ST1722 | IS609, ISEc44, ISSen6, ISRor6, | PHAGE_Entero_SfI_NC_027339(28) |
| P165 | Patient | ST3489 | MITEEc1, IS621, IS3H, IS3F | None |
| P105 | Patient | ST10 | IS100kyp, IS100X, IS100L,IS100 | PHAGE_Salmon_SEN34_NC_028699(23), PHAGE_Entero_cdtI_NC_009514(15), PHAGE_Escher_RCS47_NC_042128(53) |
| P159 | Patient | ST617 | IS3, ISEc17, IS3F, IS3H | PHAGE_Salmon_SP_004_NC_021774(19), PHAGE_Entero_BP_4795_NC_004813(9) |
| P166 | Patient | ST13846 | IS621, IS1062, ISRj1, ISAdh1 | PHAGE_Entero_mEp460_NC_019716(21 |
| P128 | Patient | ST13846 | MITEEc1, IS3H, IS3F, IS621 | PHAGE_Entero_mEp460_NC_019716(21) |
| P115 | Patient | Unknown | ISRaq1, ISSpr2, ISKpn34, ISKpn78, ISKpn80, IS1222, ISSen4, ISEhe4, ISYen3 | PHAGE_Salmon_SSU5_NC_018843(42), PHAGE_Pseudo_phiPSA1_NC_024365(7), PHAGE_Entero_mEp460_NC_019716(16), PHAGE_Entero_BP_4795_NC_004813(7) |
| P143 | Patient | Unknown | IS1396, ISAtsp1, IS1090, IS231K | PHAGE_Entero_lambda_NC_001416(21), PHAGE_Entero_mEp460_NC_019716(15) |
| P63 | Patient | Unknown | ISEhe4, ISKpn80, ISKpn34, ISRaq1 | PHAGE_Entero_IME10_NC_019501(13), |
| P72 | Patient | ST167 | MITEEc1, IS621, MiTEYpe1, Tn4430 | None |
| E37 | Environment | ST410 | IS609 | None |
| E29 | Environment | ST450 | IS2, ISEc27 | PHAGE_Entero_fiAA91_ss_NC_022750(26), PHAGE_Escher_phiV10_NC_007804(40), PHAGE_Stx2_c_Stx2a_F451_NC_049924(3), PHAGE_Pseudo_phiPSA1_NC_024365(7) |
| E25B | Environment | ST127 | ISEc42, MITEEc1, ISPa18, IS621 | PHAGE_Salmon_SEN34_NC_028699(24), PHAGE_Shigel_SfII_NC_021857(32), PHAGE_Entero_mEp460_NC_019716(26), PHAGE_Entero_I2_2_NC_001332(8) |
| E53 | Environment | ST648 | IS621, ISRor3, ISHne4, ISPa126 | PHAGE_Escher_TL_2011b_NC_019445(42), PHAGE_Klebsi_4LV2017_NC_047818(28) |
| E50-1 | Environment | ST155 | IS609, ISEc38, ISEc13, ISEc44 | None |
| E55-2 | Environment | ST58 | MITEEc1, MITEKpn1, MITEYpe1, IS621 | PHAGE_Yersin_L_413C_NC_004745(23), PHAGE_Salmon_118970_sal3_NC_031940(4), PHAGE_Entero_mEp460_NC_019716(20) |
| E56 | Environment | Unknown | IS621, ISHch13, ISHce1, ISCosp3 | PHAGE_Escher_500465_1_NC_049342(36), PHAGE_Entero_cdtI_NC_009514(5) |
